# Silicon Nitride Deposition for Flexible Organic Electronic Devices by VHF (162 MHz)-PECVD Using a Multi-Tile Push-Pull Plasma Source

**DOI:** 10.1038/s41598-017-14122-4

**Published:** 2017-10-19

**Authors:** Ki Seok Kim, Ki Hyun Kim, You Jin Ji, Jin Woo Park, Jae Hee Shin, Albert Rogers Ellingboe, Geun Young Yeom

**Affiliations:** 1 School of Advanced Materials Science and Engineering, Sungkyunkwan University, 2066 Seobu-ro, Jangan-gu, Suwon-si, Gyeonggi-do, 16419 Republic of Korea; 2 SKKU Advanced Institute of Nano Technology (SAINT), Sungkyunkwan University, 2066 Seobu-ro, Jangan-gu, Suwon-si, Gyeonggi-do, 16419 Republic of Korea; 3 Plasma Research Laboratory, School of Physical Sciences, Dublin City University, Dublin, 9 Ireland

## Abstract

Depositing a barrier film for moisture protection without damage at a low temperature is one of the most important steps for organic-based electronic devices. In this study, the authors investigated depositing thin, high-quality SiN_x_ film on organic-based electronic devices, specifically, very high-frequency (162 MHz) plasma-enhanced chemical vapor deposition (VHF-PECVD) using a multi-tile push-pull plasma source with a gas mixture of NH_3_/SiH_4_ at a low temperature of 80 °C. The thin deposited SiN_x_ film exhibited excellent properties in the stoichiometry, chemical bonding, stress, and step coverage. Thin film quality and plasma damage were investigated by the water vapor transmission rate (WVTR) and by electrical characteristics of organic light-emitting diode (OLED) devices deposited with SiN_x_, respectively. The thin deposited SiN_x_ film exhibited a low WVTR of 4.39 × 10^−4^ g (m^2^ · day)^−1^ for a single thin (430 nm thick) film SiN_x_ and the electrical characteristics of OLED devices before and after the thin SiN_x_ film deposition on the devices did not change, which indicated no electrical damage during the deposition of SiN_x_ on the OLED device.

## Introduction

Organic-based electronic devices such as flexible organic light-emitting diodes (OLEDs) are very vulnerable to moisture and oxygen because of the formation of dark spots, which detrimentally affect the organic layer and thus significantly degrade the performance and lifetime of the devices. Therefore, high-quality thin film passivation is an essential technique in flexible organic electronic devices at low temperatures^1–3^.

Previously, the most common method for preventing the moisture and oxygen was encapsulation using glass or metal lids. However, this method is not suitable for large areas or for flexible or transparent organic devices^4–6^. For thin film passivation on flexible substrates such as polyethylene terephthalate (PET), polyethylene naphthalate (PEN), and polyethersulfone (PES), thin silicon nitride (Si_3_N_4_) film is one of the most widely used materials for effective diffusion barriers because of its excellent properties such as thermal stability, low moisture diffusion, high friction resistance, and high transparency in the visible region^7,8^. Depositing Si_3_N_4_ film with high density, good uniformity, and good adhesion on flexible substrates is important but very challenging at low temperatures^9,10^.

Many researchers have investigated depositing low-temperature Si_3_N_4_ using physical vapor deposition (PVD), atomic layer deposition (ALD), and plasma-enhanced chemical vapor deposition (PECVD)^11–13^. Among these methods, PECVD has attracted considerable attention because of its high throughput at low temperatures and good adhesion on flexible substrates. However, conventional PECVD has damage issues caused by ion bombardment during the deposition as well as issues related to the porosity and unconformal step coverage on patterned substrates^14,15^.

To resolve these problems, VHF (>30 MHz)-PECVD has been studied. The characteristics of VHF plasmas are low electron temperature, high vibration temperature, and low ion bombardment damage in addition to higher plasma than that of conventional rf (3–30 MHz) plasmas^16–23^. However, VHF causes plasma non-uniformity due to increased standing wave effects at high frequencies^24^.

In our previous studies, to minimize the standing wave effects and to remove rf current flowing through the substrates, we investigated a multi-tile push-pull (a power electrode composed of electrically floated small electrodes) plasma source and applied it to VHF plasma^25,26^. In this study, we used VHF (162 MHz)-PECVD using a multi-tile push-pull plasma source to deposit thin, high-quality SiN_x_ film, and using this source, we obtained a low WVTR of 4.39 × 10^−4^ g (m^2^ · day)^−1^ with a single thin (430 nm thick) SiN_x_ film at a low temperature of 80 °C. Furthermore, we observed no change in the electrical characteristics of the OLED devices after we deposited the thin SiN_x_ films on the OLED devices.

## Methods

### VHF (162 MHz) plasma equipment and deposition

**Figure 1:**
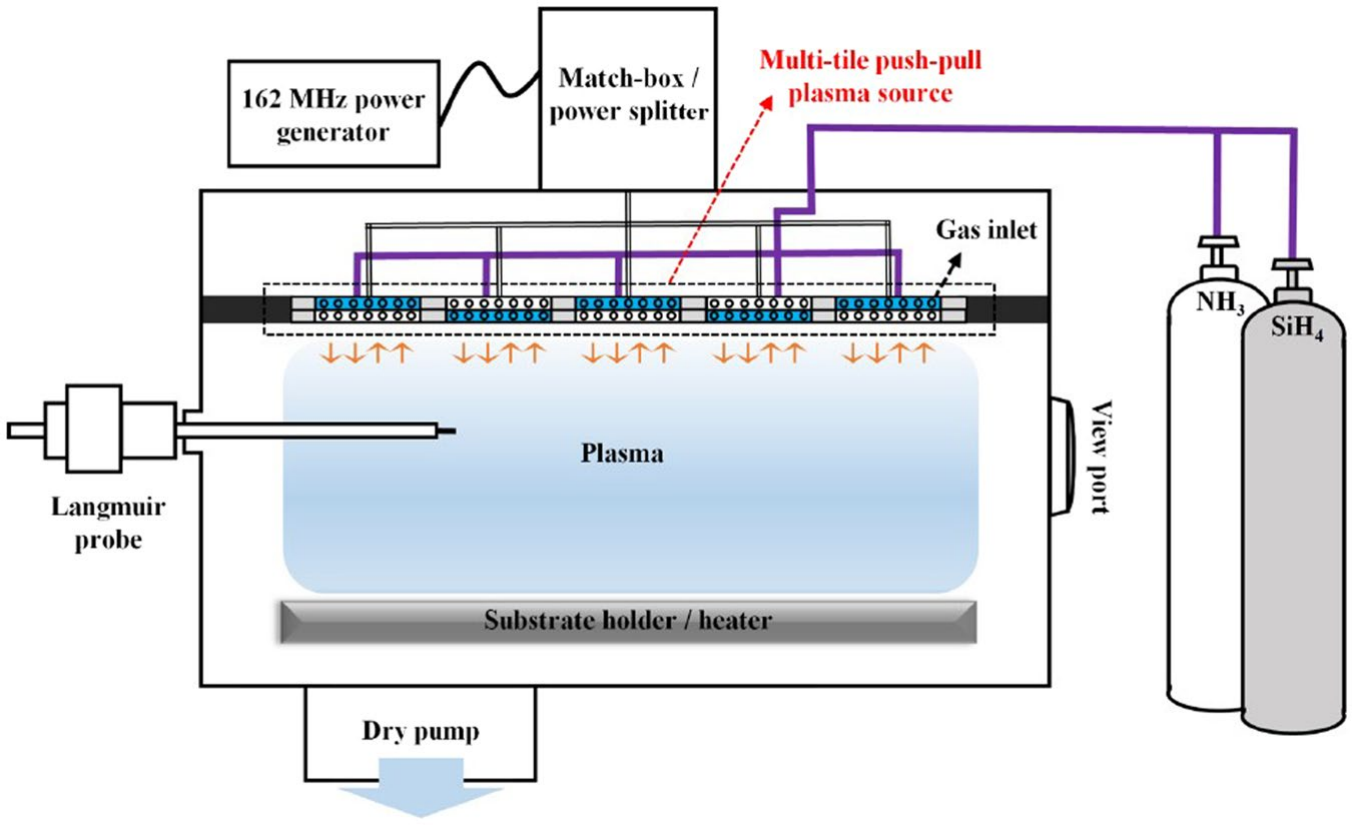
Schematic diagram. The VHF (162 MHz)-PECVD system equipped with a multi-tile push-pull plasma source used to deposit high quality SiN_x_ films.

Figure 1 shows the schematic diagram of VHF (162 MHz)-PECVD using multi-tile push-pull plasma. The multi-tile push-pull electrodes consist of five pairs of electrically floating electrodes, and each pair comprises two adjacent push-pull (alternating + /− voltage) electrodes. These electrodes were connected with a matcher/power splitter, which enabled delivering equal power to each electrode pair; the details of the VHF (162 MHz)-PECVD system using the multi-tile push-pull plasma source can be found elsewhere^25^.

**Figure 2:**
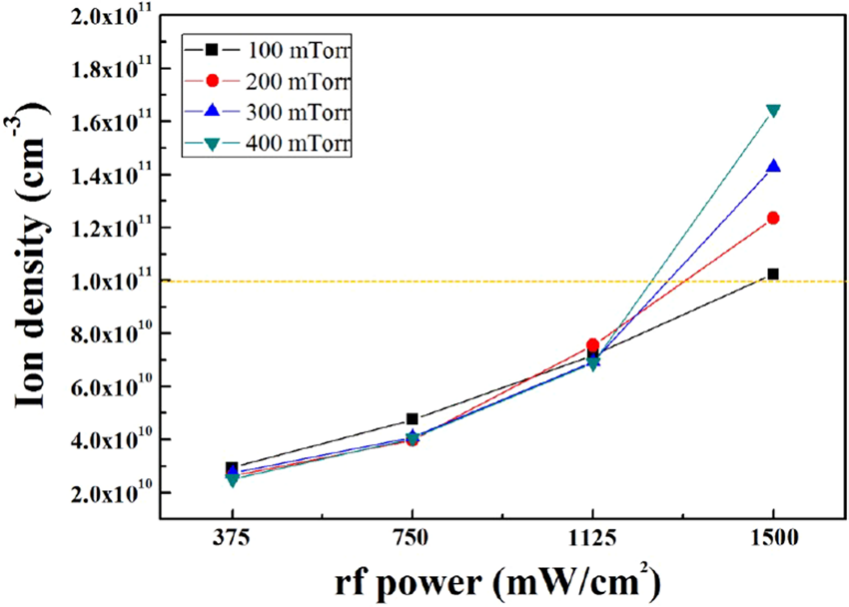
Ion density. Ion density measured using a Langmuir probe at different rf powers (375–1500 mW/cm^2^) and operating pressures (100–400 mTorr) using the 162 MHz multi-tile push-pull plasma source.

We deposited thin SiN_x_ films on soda-lime glass, silicon, and PET substrates at gas flow ratios of 100–350 sccm NH_3_/100 sccm SiH_4_, and each substrate was located 20 mm below the multi-tile push-pull electrodes. The rf power and operating pressure were maintained at 1500 mW/cm^2^ and 200 mTorr, respectively (when the operating pressure varied from 100 to 400 mTorr at 1500 mW/cm^2^, the ion density increased with pressure, and we could obtain ion density higher than 10^11^ cm^−3^ as shown in Fig. 2. However, due to the particle formation at pressures higher than 300 mTorr, we kept the operating pressure lower than 300 mTorr in our experiment). We maintained the temperature of the substrate holder at 80 °C to keep the temperature of the substrate surface lower than 80 °C because the glass transition temperature of flexible substrates generally starts from 100 °C and OLED devices also degrade at temperatures higher than 100 °C^27,28^.

### OLED fabrication

We formed the OLED device by thermally evaporating organic materials on an ITO glass substrate through the following sequence: (1) 50 nm of 2-TNATA, (2) 30 nm of NPB, (3) 30 nm of Alq_3_, (4) 0.7 nm of LiF and (5) 100 nm of Al cathode (Fig. S1, Supplementary Information). We measured the electrical characteristics of the OLED devices before and after depositing the SiN_x_ films using a Spectroradiometer (CS-2000, Konica Minolta).

### Characterization

We investigated the plasma characteristics by measuring ion density using a Langmuir probe (ESP, Hiden Analytical Inc.) and estimated the uniformity of the plasma by measuring the ion saturation current using a homemade electrostatic probe. The electrostatic probe was biased at −30 V, and we measured the ion saturation current with a Keithley 2000 after passing it through an rf filter.

We investigated the chemical binding states of the thin SiN_x_ films by Fourier transform infrared spectroscopy (FTIR, Bruker IFS-66/S, TENSOR27) in the wave number range of 500–4000 cm^−1^. We measured the stoichiometry of the films by X-ray photoelectron spectroscopy (XPS, MultiLab 2000, Thermo VG, Mg Kα source) after we calibrated the peak energies based on C1s peak at 284.5 eV. We measured the optical transmittance of the thin films by ultraviolet-visible near infrared (UV-Vis NIR) spectroscopy (Shimadzu, UV-3600), and we observed the step coverage and density of the films by field-emission scanning electron microscopy (FE-SEM, Hitachi S-4700). We conducted the WVTR test of the thin SiN_x_ films using a coulometric detector (Aquatran Model 2, Mocon Inc.) at the 40 °C and 100% relative humidity; we measured the stress of the films (FSM500TC, Frontier Semiconductor) in the dual wavelength (780 and 650 nm).

## Results and Discussion

Figure 2 shows the ion density measured using a Langmuir probe at different rf powers (375–1500 mW/cm^2^) and operating pressures (100–400 mTorr) for the 162 MHz multi-tile push-pull plasma source. The plasma density increased gradually with increasing rf power, but we observed no significant increase with increasing operating pressure until it reached 1125 mW/cm^2^. At the rf power of 1500 mW/cm^2^, we obtained plasma density of 1.0 × 10^11^ cm^−3^ or higher under all pressure conditions, whereas conventional HF (13.56 MHz) capacitively coupled plasma systems generally show low plasma densities less than 1.0 × 10^10^ cm^−3^ because plasma density is proportional to operating frequency [f^3/2^]^29^.

**Figure 3:**
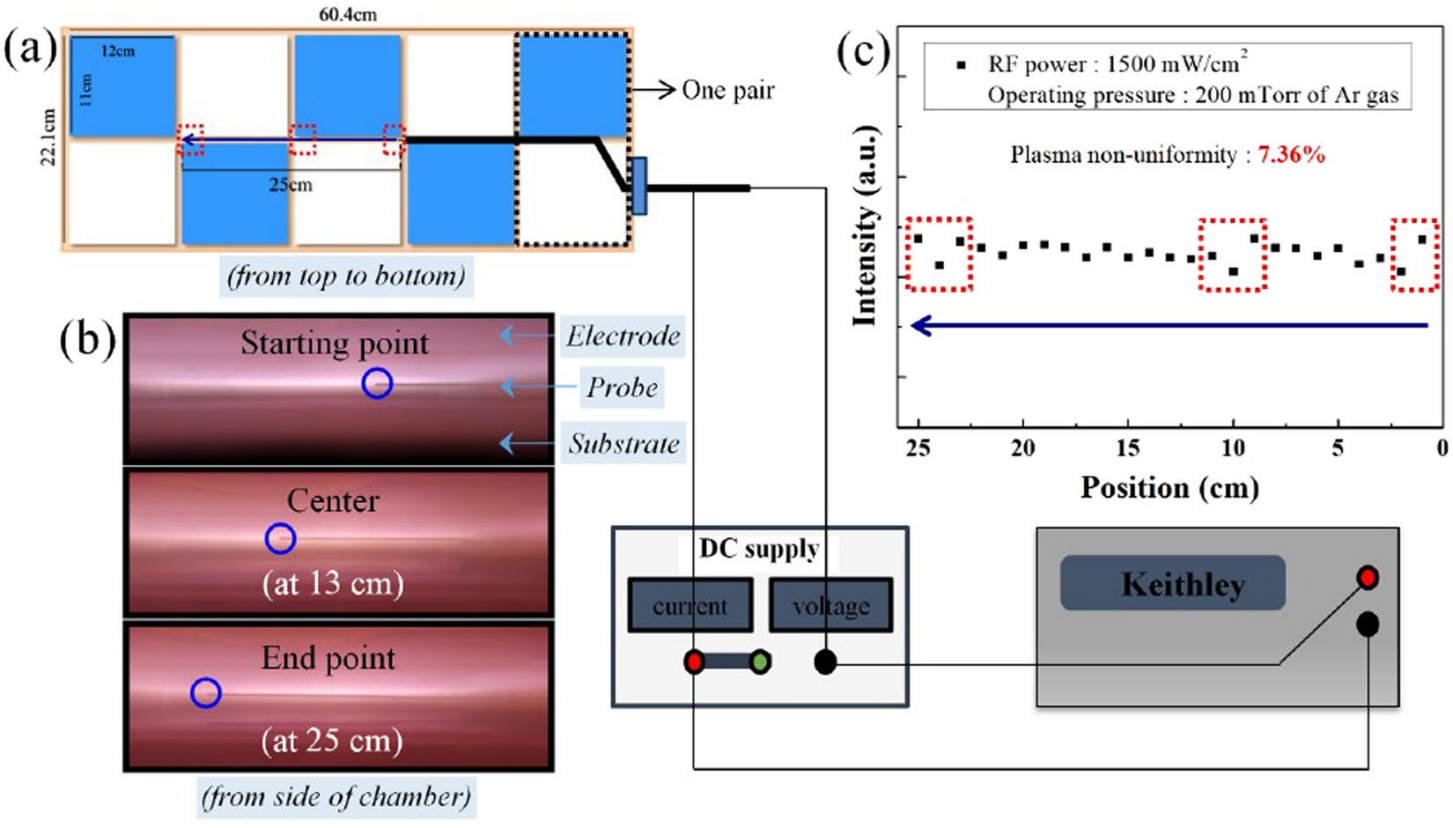
Plasma uniformity. (**a**) Schematic diagram of the multi-tile push-pull electrodes in the deposition system and an electrostatic probe installed in the system; (**b**) Plasma images near the electrodes showing the measurement range of the ion saturation current using the electrostatic probe; (**c**) Uniformity of the ion saturation current along the centerline of the multi-tile push-pull electrodes.

To confirm the presence of a standing wave effect due to the VHF of 162 MHz, we measured the plasma uniformity between the multi-tile push-pull electrodes; Fig. 3(a) shows the schematic diagram of the multi-tile push-pull electrodes (one blue tile and one white tile are one push-pull electrode pair) and an electrostatic probe installed between the tiles in the system. We installed the probe 20 mm below the multi-tile push-pull electrode surface and maintained the rf power and operating pressure at 1500 mW/cm^2^ and 200 mTorr of Ar gas, respectively. The electrostatic probe was biased at −30 V to measure the ion saturation. We measured the plasma uniformity along the center line, shown as red squares in Fig. 3(a), by 25 points with 1 cm interval; the measured points are also shown in Fig. 3(b), and we observed them from the side of the multi-tile electrodes during the operation of the VHF plasma. As shown in Fig. 3(c), the plasma non-uniformity estimated by the ion saturation current measured in a high-density plasma region (>10^11^ cm^−3^) was approximately 7.36%, reflecting that the plasma was relatively uniform. Also, due to the relatively small electrode sizes of multi-tile electrodes, we observed no uniformity issues caused by the standing wave effect, although we did note low uniformity at the edges of the tiles as denoted by the red squares in Fig. 3(a),(b); we believed that this low plasma uniformity at the edge was caused by our disturbing the electric field and that this issue may need to be resolved in the near future.

**Figure 4:**
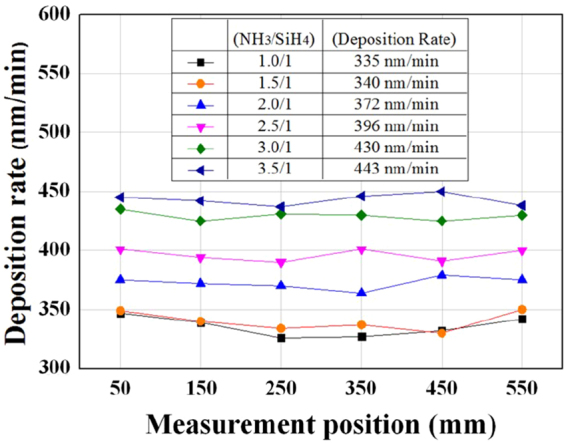
Deposition rate of thin SiN_x_ films deposited along the centerline of the multi-tile push-pull electrodes at 1500 mW/cm^2^, 200 mTorr, and 80 °C substrate temperature varying gas flow rates from 100 to 350 sccm NH_3_/100 sccm SiH_4_. Six points were measured at 100-mm intervals.

Using the plasma conditions in Fig. 3, we deposited the thin SiN_x_ films with NH_3_/SiH_4_. Figure 4 shows the films’ deposition rates deposited along the centerline at 1500 mW/cm^2^, 200 mTorr, and 80 °C substrate temperature with gas flow rates varying from 100 to 350 sccm NH_3_/100 sccm SiH_4_. To measure the deposition rate, we loaded 4-inch silicon wafers at 100 mm intervals at the center of the push-pull electrodes and calculated the averages of the five points of each wafer. As shown in Fig. 4, as the gas mixture ratio of (NH_3_/SiH_4_) increased from 1.0/1 to 3.5/1, the average deposition rate of the thin SiN_x_ films increased gradually from 335 nm/min to 443 nm/min.

**Figure 5:**
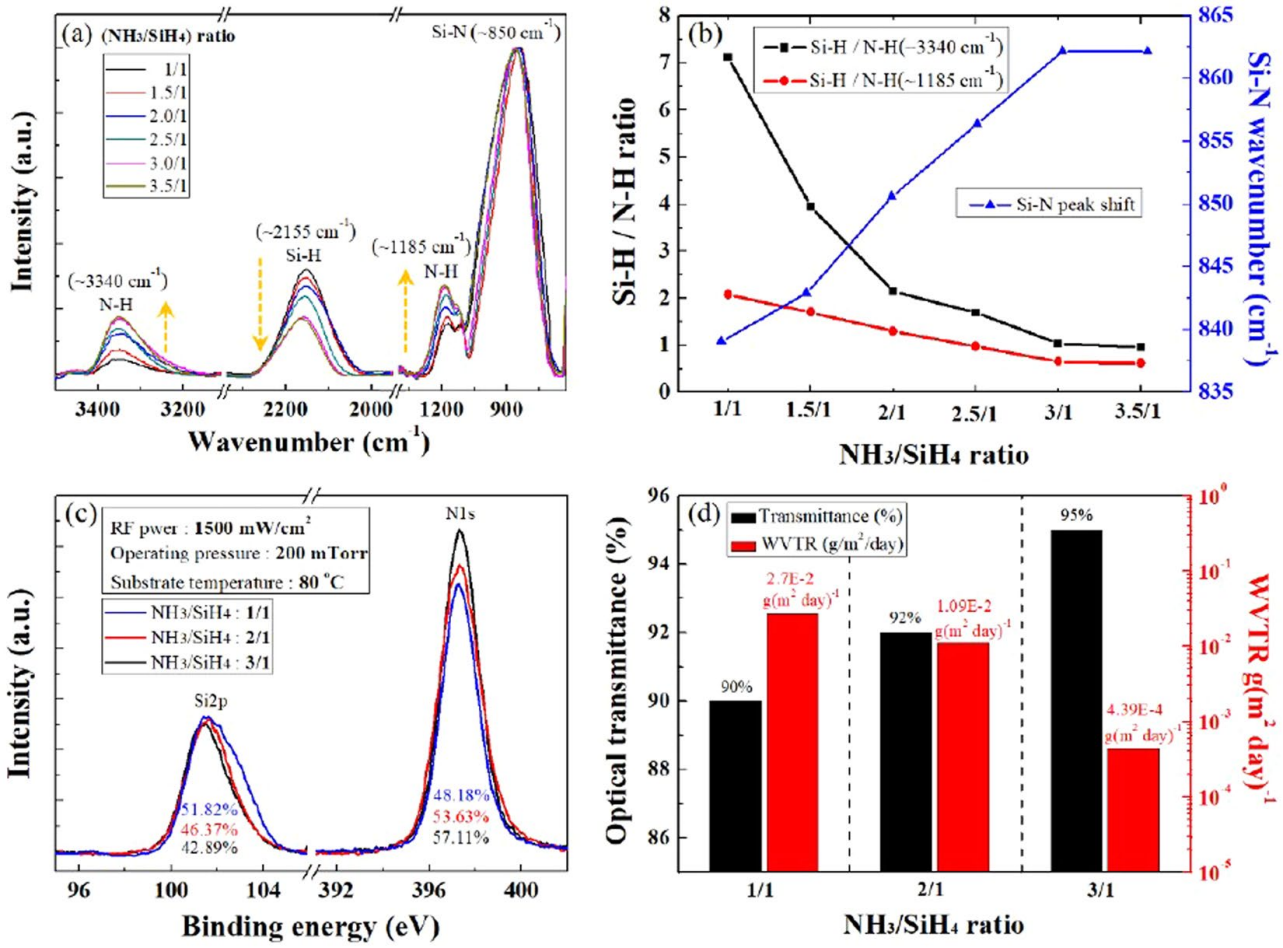
Physical properties of thin SiN_x_ films. Characteristics of thin SiN_x_ films measured by FTIR, XPS, UV-Vis NIR spectroscopy, and WVTR test at NH_3_/SiH_4_ gas mixture ratios of 1–3.5/1; (**a**) FTIR spectra of 335- to 430-nm thick SiN_x_ films deposited at NH_3_/SiH_4_ ratios of 1–3.5/1; (**b**) Absorption peak intensities ratios of Si-H/N-H and the absorption peak positions of Si-N extracted from the FTIR spectra of (**a**); (**c**) XPS narrow scan data of Si 2p at 101 eV and N 1 s at 398 eV in the thin SiN_x_ films at the NH_3_/SiH_4_ ratios of 1/1, 2/1 and 3/1, respectively; (**d**) Optical transmittances measured using UV-Vis NIR spectroscopy and the WVTR test for the three conditions in (**c**).

Figure 5 show the characteristics of the thin SiN_x_ films measured by FTIR, XPS, UV-Vis NIR spectroscopy, and WVTR test at gas mixture ratios of 1–3.5/1 (NH_3_/SiH_4_), as shown in Fig. 4. Figure 5(a) shows the FTIR spectra of 335- to 430-nm thick films deposited at the different gas mixture ratios. We observed N-H stretching (approximately 3340 and 1185 cm^−1^), Si-H stretching (approximately 2155 cm^−1^), and Si-N stretching (approximately 850 cm^−1^) at a wave number range of 600–3500 cm^−1^. To analyze the change of the peak intensities related to N-H bonding and Si-H bonding, we normalized the Si-N absorption peak; Fig. 5(b) shows the absorption peak intensity ratios of Si-H/N-H and the peak positions of Si-N extracted from the FTIR spectra in Fig. 5(a). As the gas mixture ratios of NH_3_/SiH_4_ increased from 1/1 to 3/1, the ratios of Si-H/N-H (~3340 cm^−1^) and Si-H/N-H (~1185 cm^−1^) decreased gradually, indicating more nitrogen in the deposited film. We also observed a gradual shift of Si-N absorption peak position from 839 cm^−1^ to 862 cm^−1^ with the increase of the NH_3_/SiH_4_ ratio, and we believed this was related to the increased film density with increasing nitrogen in the film because the Si-H bond is longer (150 pm) than the N-H bond (101 pm); therefore, the decreased Si-H/N-H ratio in the film may indicate increased film density^30^ even though previous studies showed that increased N/Si in the film increases SiN_x_ density^31^. When we increased the gas mixture ratio from 3.0/1 to 3.5/1 (NH_3_/SiH_4_), we observed no changes in either Si-H/N-H ratios or the Si-N absorption peak shift, indicating that the SiN_x_ film was saturated with nitrogen. We also measured stress measurement to confirm the mechanical properties of the thin SiN_x_ film. As the NH_3_/SiH_4_ gas mixture ratios increased from 1.0/1 to 3.0/1, the stress on the film decreased gradually from 117 to 21.94 (MPa), and at the NH_3_/SiH_4_ ratio of 3.5/1, the stress on the film was almost saturated (Fig. S2, Supplementary Information). Figure 5(c) shows the XPS narrow scan data of Si 2p at 101 eV and N 1 s at 398 eV in the thin SiN_x_ films at the gas mixture ratios of 1.0/1, 2.0/1, and 3.0/1 (NH_3_/SiH_4_), respectively. The atomic percentage ratio (N/Si) of the deposited films (ignoring their H content) increased from 0.93 (N/Si = 48.18%/51.82%) to 1.33 (N/Si = 57.11%/42.89%) as the gas mixture ratio of NH_3_/SiH_4_ increased from 1.0/1 to 3.0/1; therefore, the composition of the thin SiN_x_ film changed from silicon-rich SiN_x_ film to stoichiometric amorphous Si_3_N_4_. In addition, as shown in Fig. 5(d), as the N/Si ratio increased from 0.93 to 1.33 by increasing the NH_3_/SiH_4_ ratio from 1.0/1 to 3.0/1, the optical transmittance increased from 90 to 95% and the WVTR decreased from 2.7 × 10^−2^ to 4.39 × 10^−4^ g (m^2^ · day)^−1^ (even though the SiN_x_ for NH_3_/SiH_4_ of 1.0/1 was thinner, 335 nm, than it was at 3.0/1, 443 nm, the quality of SiN_x_ determined the WVTR value more significantly than the thickness). As a result, we confirmed that using the VHF (162 MHz) multi-tile push-pull plasma source, we could obtain a high-quality, stoichiometric thin Si_3_N_4_ film with high optical transmittance and a low WVTR at a high deposition rate (430 nm/min) and a low deposition temperature (80 °C).

**Figure 6:**
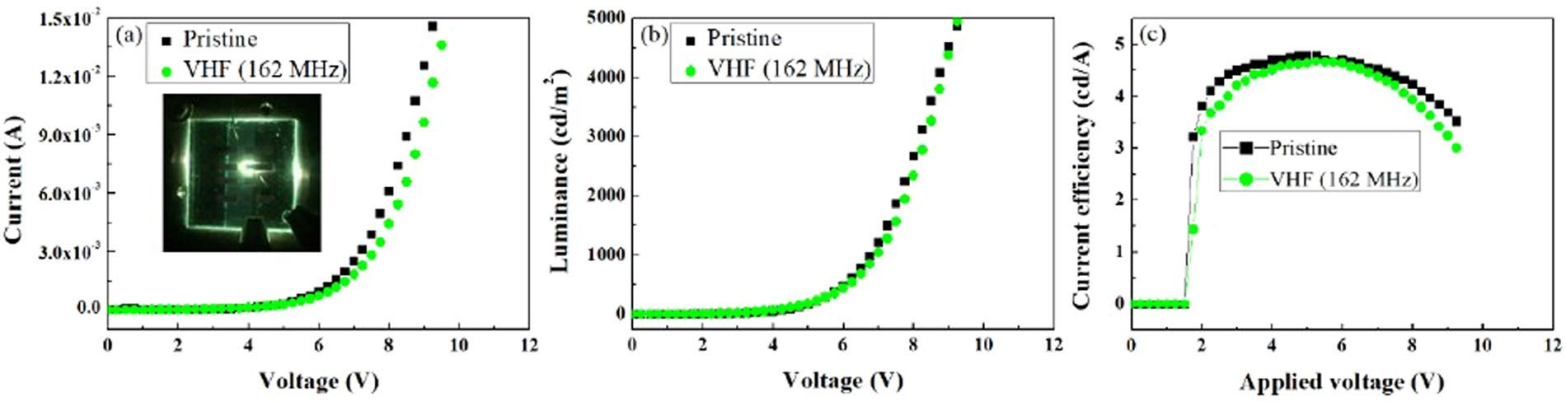
Analysis of OLED device damage before and after thin SiN_x_ film deposition. (**a**) I-V, (**b**) L-V, and (**c**) current efficiency-voltage characteristics before and after the films were deposited on the devices by VHF (162 MHz)-PECVD using the multi-tile push-pull plasma source.

Figure 6 shows the electrical characteristics of the OLED devices measured before and after we deposited the thin SiN_x_ films on the devices. Specifically, we compared the films deposited by VHF (162 MHz)-PECVD using the multi-tile push-pull plasma source and conventional PECVD operated at 13.56 MHz under the same process conditions (1500 mW/cm^2^ of rf power, 1 min of operating time, 200 mTorr of operating pressure, NH_3_/SiH_4_ gas mixture ratio of 3/1, and substrate temperature of 80 °C; Fig. S3, Supplementary Information). Figure 6(a),(b) show the current-voltage (I-V) and luminance-voltage (L-V) characteristics before and after we deposited the 430-nm thick SiN_x_ thin film on the OLED devices by VHF (162 MHz)-PECVD using the multi-tile push-pull plasma source, respectively, and the figures show similar electrical characteristics. The inset in Fig. 6(a) shows the green light emitted from the OLED device after the deposition of the thin SiN_x_ film on the OLED devices by multi-tile push-pull VHF (162 MHz)-PECVD. Figure 6(c) shows the current efficiency-voltage characteristics before and after we deposited the films on the OLED devices by VHF (162 MHz)-PECVD and the current efficiency at 5.25 V was 4.76 and 4.68 (cd/A) before and after the film deposition, indicating almost no electrical damage to the organic layer of the OLED device after the SiN_x_ deposition. In contrast, as shown in Fig. S3 (#3), when we deposited 310-nm film on the device by conventional PECVD at 13.56 MHz, the turn-on voltage increased, and we observed unstable electrical characteristics after we deposited the film on the device (Samples #1, #2, and #4 are reasonable thin SiN_x_ film deposition conditions at conventional PECVD (13.56 MHz), and #3 is the same condition as the film deposition at VHF (162 MHz)-PECVD on the device). These results show that VHF (162 MHz)-PECVD using a multi-tile push-pull plasma source can deposit a high-quality thin SiN_x_ film without damaging an OLED device due to the low electron temperature and lack of rf current flow to the substrate by using electrically floated push-pull electrode pairs as observed in previous studies^25,26^. In addition, we confirmed excellent step coverage and thin SiN_x_ film quality with VHF (162 MHz)-PECVD using a multi-tile push-pull plasma source, in contrast with the conventional PECVD operated at 13.56 MHz (Fig. S4, Supplementary Information).

## Conclusions

In this study, we were able to form high-density, uniform plasmas without the standing wave effect for VHF of 162 MHz by using a multi-tile push-pull plasma source. In addition, using VHF-PECVD, we could deposit stoichiometric Si_3_N_4_ with a high deposition rate (430 nm/min) under the optimized process conditions of 1500 mW/cm^2^ of rf power, 200 mTorr of operating pressure, gas mixture ratio of 3/1 (NH_3_/SiH_4_), and 80 °C substrate temperature. Under these conditions, the deposited 430-nm thick SiN_x_ film exhibited a high optical transmittance of 95% and a low WVTR of 4.39 × 10^−4^ g (m^2^ · day)^−1^. Finally, when we deposited the thin SiN_x_ films on OLED devices and patterned samples, we observed no electrical damage to devices and more step coverage on the patterned samples with the multi-tile push-pull VHF (162 MHz)-PECVD compared with conventional PECVD operated at 13.56 MHz. We believe that VHF (162 MHz)-PECVD thin film deposition using a multi-tile push-pull plasma source can be applied not only to thin Si_3_N_4_ film deposition for flexible organic electronic devices but also to PECVD deposition of various next-generation semiconductor materials by requiring high-quality materials at high deposition rates.

## Electronic supplementary material

Supplementary Information
