# Supplementary material for: Silicon Nitride Deposition for Flexible Organic Electronic Devices by VHF (162 MHz)-PECVD Using a Multi-Tile Push-Pull Plasma Source
Source: Sci Rep. 2017 Oct 19;7:13585. doi: 10.1038/s41598-017-14122-4 (PMC5648850; doi:10.1038/s41598-017-14122-4)
Supplement: Supplementary file 1 — Supplementary Information [file 41598_2017_14122_MOESM1_ESM.pdf]

Supplementary Information

Silicon Nitride Deposition for Flexible Organic Electronic Devices by VHF (162 MHz)-PECVD Using a Multi-Tile Push-Pull Plasma Source

Ki Seok Kim [1,‡], Ki Hyun Kim [1,‡], You Jin Ji [1], Jin Woo Park [1], Jae Hee Shin [3], Albert Rogers Ellingboe [3], and Geun Young Yeom [1,2,*]

[1.] *School of Advanced Materials Science and Engineering, Sungkyunkwan University, 2066 Seobu-ro, Jangan-gu, Suwon-si, Gyeonggi-do 16419, Republic of Korea.*
[2.] *SKKU Advanced Institute of Nano Technology (SAINT), Sungkyunkwan University, 2066 Seobu-ro, Jangan-gu, Suwon-si, Gyeonggi-do 16419, Republic of Korea.*
[3.] *Plasma Research Laboratory, School of Physical Sciences, Dublin City University, Dublin 9, Ireland.*

[‡]These authors contributed equally to this work
*Corresponding Author : gyyeom@skku.edu

Schematic diagram of OLED device using organic materials

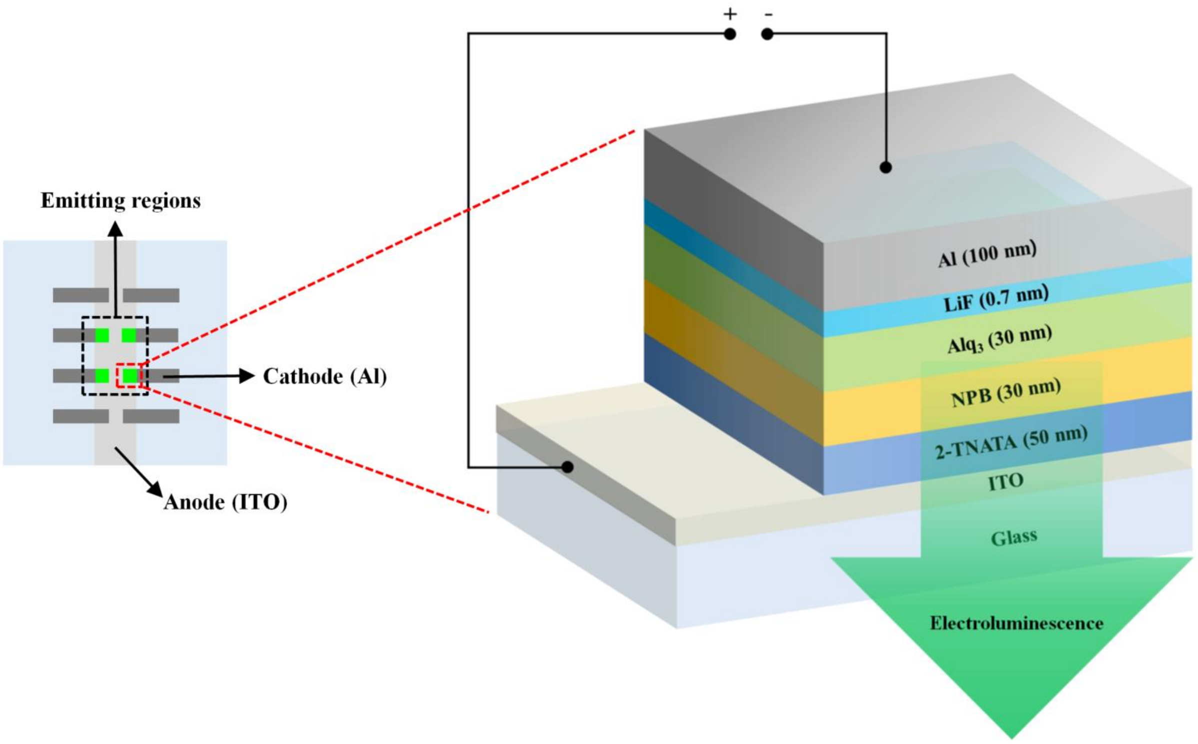

Figure S1. The schematic diagram of the OLED device and the order of the deposited organic materials using thermal evaporation.

Stress of thin SiN_x films deposited by VHF (162 MHz)-PECVD

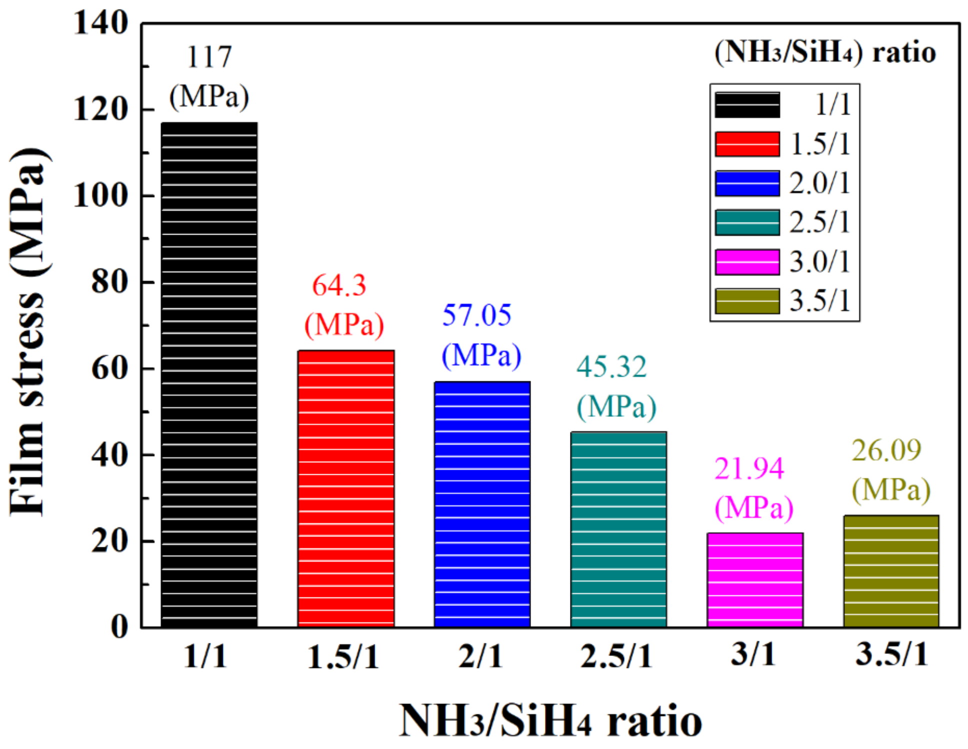

Figure S2. Stress analysis of thin SiN_x films deposited with NH_3/SiH_4 gas mixture ratios of 1.0–3.5/1 in VHF (162 MHz)-PECVD using the multi-tile push-pull plasma source.

I-V characteristics of the OLED devices measured before and after the deposition of the thin SiN_x films by conventional PECVD operated at 13.56 MHz

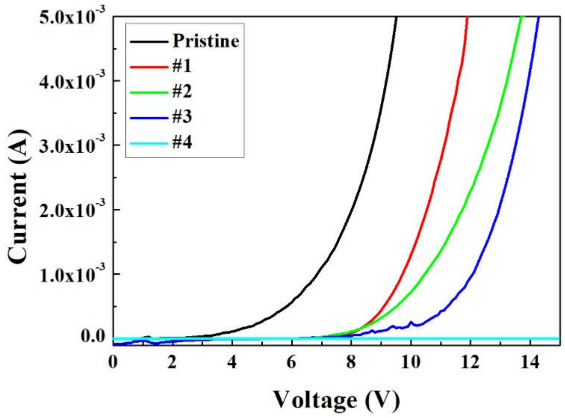

	Sample #	RF power [mW/cm^2]	SiH_4 [sccm]	NH_3 [sccm]	Pressure [mTorr]
HF (13.56 MHz) Conventional PECVD	#1	750	100	90	100
	#2	1125	100	90	100
	#3	1500	100	300	200
	#4	1500	100	90	100

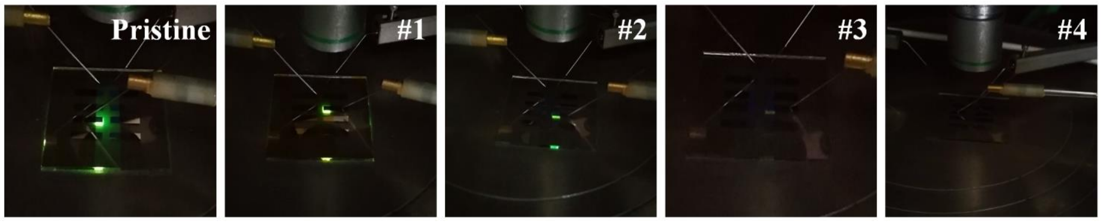

Figure S3. Analysis of OLED device damage after film deposition for 1 min under various process conditions by conventional PECVD operated at 13.56 MHz.

Step coverages of thin SiN_x films deposited by VHF (162 MHz)-PECVD and conventional PECVD operated at 13.56 MHz

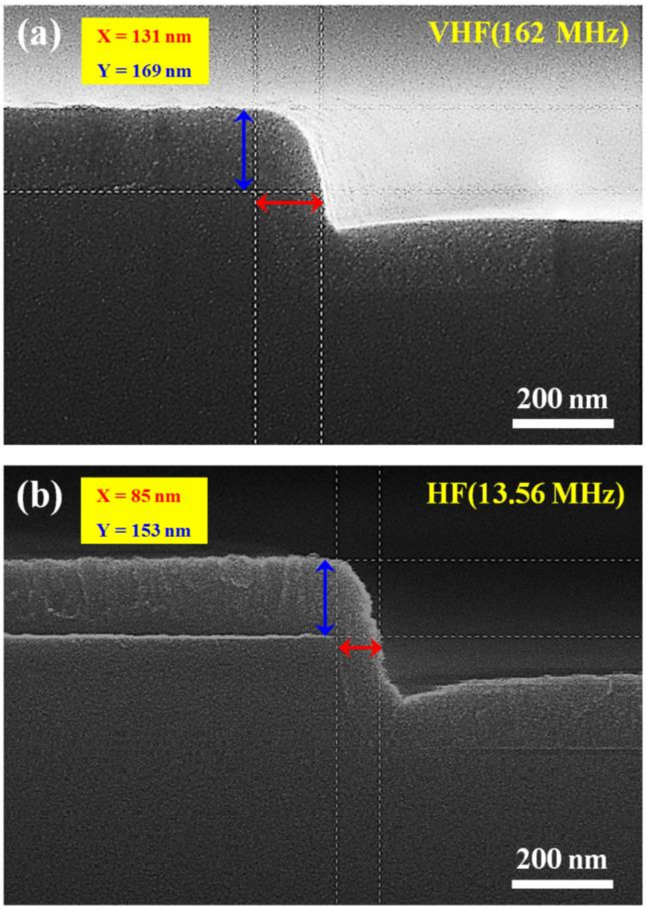

Figure S4. Step coverage analysis of a thin SiN_x film deposited on a patterned sample under the same conditions by (**a**) VHF (162 MHz)-PECVD using a multi-tile push-pull plasma source and (**b**) conventional PECVD operated at 13.56 MHz (HF).

We deposited thin SiN_x films were deposited on patterned samples using the VHF (162 MHz) multi-tile push-pull plasma source and a conventional capacitively coupled plasma source operated at 13.56 MHz (to deposit the films for step coverage, we used patterned silicon samples rather than OLED samples. Here, the step height of OLED device used in our experiment was about 211 nm with sloped profiles, whereas the step height of the patterned silicon sample was about 240 nm with a more vertical profile. Therefore, it is believed that there is no problem in comparing the characteristics of the step coverage). The deposition conditions for VHF (162 MHz)-PECVD and conventional PECVD operated at 13.56 MHz were the same as in Figure 6, but the deposition times were 25 and 30 s,

respectively, for the comparison of the step coverage with similar SiN_x film thicknesses. As shown in the figures, the step coverage of the thin films deposited with the VHF (162 MHz)-PECVD and the conventional PECVD operated at 13.56 MHz were 77.5 and 55.5%, respectively. The SiN_x deposited by VHF (162 MHz)-PECVD exhibited better step coverage than that by conventional PECVD at 13.56 MHz, and the film quality estimated by the SEM profile was also better. We believed these better characteristics to be related to higher vibration temperatures of the dissociated species for the VHF (162 MHz) plasma using multi-tile push-pull electrodes[1].

References

1. Kim, K. S. *et al*. Characteristics of silicon nitride deposited by VHF (162 MHz)-plasma enhanced chemical vapor deposition using a multi-tile push–pull plasma source. *J. Phys. D: Appl. Phys.* **49**, 395201 (2016).
